# Differential features of chronic cough according to etiology and the simple decision tree for predicting causes

**DOI:** 10.1038/s41598-021-89741-z

**Published:** 2021-05-14

**Authors:** Hyeon-Kyoung Koo, Won Bae, Ji-Yong Moon, Hyun Lee, Jin Woo Kim, Seung Hun Jang, Hyoung Kyu Yoon, Deog Kyeom Kim

**Affiliations:** 1 Division of Pulmonary and Critical Care Medicine, Department of Internal Medicine, Ilsan Paik Hospital, Inje University College of Medicine, Ilsan, Republic of Korea; 2 Department of Internal Medicine, Hanyang University College of Medicine, Guri, Republic of Korea; 3 Department of Internal Medicine, Hanyang University College of Medicine, Seoul, Republic of Korea; 4 Division of Pulmonary and Critical Care Medicine, Department of Internal Medicine, Uijeongbu St. Mary’s Hospital, College of Medicine, The Catholic University of Korea, Uijeongbu, Republic of Korea; 5 Division of Pulmonary, Allergy, and Critical Care Medicine, Department of Medicine, Hallym University Sacred Heart Hospital, Hallym University College of Medicine, Anyang, Republic of Korea; 6 Division of Pulmonary and Critical Care Medicine, Department of Internal Medicine, Yeouido St. Mary’s Hospital, College of Medicine, The Catholic University of Korea, Seoul, Republic of Korea; 7 Division of Pulmonary and Critical Care Medicine, Department of Internal Medicine, Seoul Metropolitan Government-Seoul National University Boramae Medical Center, Seoul National University College of Medicine, Seoul, Republic of Korea

**Keywords:** Medical research, Signs and symptoms

## Abstract

Finding etiology of chronic cough is an essential part of treatment. Although guidelines include many laboratory tests for diagnosis, these are not possible in many primary care centers. We aimed to identify the characteristics and the differences associated with its cause to develop a clinical prediction model. Adult subjects with chronic cough who completed both Korean version of the Leicester Cough Questionnaire (K-LCQ) and COugh Assessment Test (COAT) were enrolled. Clinical characteristics of each etiology were compared using features included in questionnaires. Decision tree models were built to classify the causes. A total of 246 subjects were included for analysis. Subjects with asthma including cough variant asthma (CVA) suffered from more severe cough in physical and psychological domains. Subjects with eosinophilic bronchitis (EB) presented less severe cough in physical domain. Those with gastro-esophageal reflux disease (GERD) displayed less severe cough in all 3 domains. In logistic regression, voice hoarseness was an independent feature of upper airway cough syndrome (UACS), whereas female sex, tiredness, and hypersensitivity to irritants were predictors of asthma/CVA; less hoarseness was a significant feature of EB, and feeling fed-up and hoarseness were less common characteristics of GERD. The decision tree was built to classify the causes and the accuracy was relatively high for both K-LCQ and COAT, except for UACS. Voice hoarseness, degree of tiredness, hypersensitivity to irritants and feeling fed-up are important features in determining the etiologies. The decision tree may further assists classifying the causes of chronic cough.

## Introduction

Cough is one of the most common symptoms of pulmonary and extra-pulmonary diseases, leading people to seek medical attention^1,2^. Though cough reflex is an important defense mechanism for airway clearance^3,4^, it is just regarded as an annoyance that impairs quality of life for many patients, especially if it persists chronically^2^. However, finding the etiology of chronic cough is not an easy process, as it can be developed through various reasons. Upper airway cough syndrome (UACS), asthma including cough variant asthma (CVA), eosinophilic bronchitis (EB), and gastroesophageal reflux syndrome (GERD) have been emphasized as the primary causes of chronic cough in non-smokers with normal chest radiographs^5–13^. Additionally, a recent study highlighted that the same causes could also be applied to smokers^14^. Based upon these causes, there have been several efforts to make an effective diagnostic algorithm. Guidelines for chronic cough usually include chest radiography, spirometry with the bronchodilator reversibility test, the bronchoprovocation test, induced sputum analysis, and gastro-endoscopy^7–13,15^. However, as these tests need several specialized laboratory devices and space, this may not be feasible among many primary care centers with limited resources. For these circumstances, some guidelines also recommend empirical treatment for possible causes first and subsequent re-assessment following the treatment response as an alternative method^16,17^. Therefore, it is essential to develop a simple algorithm to identify plausible etiology based on clinical characteristics, allowing for the treatment of chronic cough among primary clinics. The aim of this study was to describe the phenotypic characteristics of chronic cough, compare their differences according to its cause, and develop prediction models for etiology using two cough related quality of life questionnaires: the Korean version of the Leicester Cough Questionnaire (K-LCQ) and the Cough Assessment Test (COAT).

## Results

### Baseline characteristics

A total of 322 subjects with chronic cough (male 121, female 201: mean age 47.9 ± 15.1 years) were enrolled from 16 respiratory centers between December 2016 to July 2017. Among them, 246 subjects (76.4%; male 90, female 156; mean age 47.4 ± 14.1) who completed the both questionnaires with necessary diagnostic work-up were included in present analysis. Baseline characteristics of these subjects are summarized in Table 1. The mean score of COAT was 11.3 (± 4.1) and that of K-LCQ was 11.1 (± 3.2). The score of the COAT and the K-LCQ are negatively correlated and the correlation matrix between each item of the COAT and the K-LCQ is described in Supplemental Fig. S2. The median duration of cough was 12 weeks (IQR 8.0–24.0). Among them, 115 patients (46.7%) were diagnosed as UACS, 65 (26.4%) as asthma/CVA, 40 (16.3%) as EB, 31 (12.6%) as GERD, 16 (6.5%) could not find the cause, and 20 subjects (8.1%) had multiple causes. Eleven patients had both UACS and asthma/CVA, 2 patients had both UACS and EB, 6 patients had both UACS and GERD, and 3 patients had both asthma/CVA and GERD (Supplemental Fig. S3). There was no significant difference in age or cough duration before visit among these causes, but female sex was predominant in asthma/CVA (76.9%). Subjects with asthma/CVA complained of more severe degree of cough in total scores of COAT and K-LCQ, especially physical and psychological domains. On the contrary, patients with EB had less severity in physical domain of K-LCQ, and those with GERD showed less severity in COAT and all three domains of K-LCQ. Patients with UACS and idiopathic cough demonstrated no difference in COAT and K-LCQ scores (Table 1). Sex was not associated with scores of either COAT (p = 0.14) or K-LCQ among all three domains (physical, p = 0.06; psychological, p = 0.40; social, p = 0.29; total, p = 0.19). However, as age increases, scores of COAT decrease (regression coefficient =  − 0.07, p = 0.0002) and that of K-LCQ increase in both psychological (regression coefficient = 0.02, p = 0.001) and social domains (regression coefficient = 0.02, p = 0.003).

**Table 1:** Comparison of baseline characteristics and cough severity among different etiologies of chronic cough.

	Total (N = 246)	UACS (N = 115)	Asthma/CVA (N = 65)	EB (N = 40)	GERD (N = 31)	Idiopathic (N = 16)
Age, mean ± SD	47.4 ± 14.1	46.8 ± 14.6	48.3 ± 14.1	46.6 ± 13.2	50.6 ± 14.9	48.0 ± 16.4
Male sex, N (%)	90 (36.6%)	45 (39.1%)	15 (23.1%) *	14 (35.0%)	13 (41.9%)	8 (50%)
Current smoking, N (%)	12 (5.2%)	3 (2.6%)	2 (3.1%)	2 (5.0%)	5 (16.1%) *	0 (0%)
Cough duration, weak (Q1, Q3)	12.0 (8.0, 24.0)	12.0 (8.0, 20.0)	16.0 (10.0, 24.5)	13.0 (9.0, 24.0)	12.0 (10.0, 15.75)	12.0 (8.0, 24.0)
**Cough severity**						
Cough NRS	6.0 ± 2.2	6.1 ± 2.2	6.5 ± 2.2	5.7 ± 2.1	5.4 ± 2.3*	6.2 ± 2.1
COAT total	11.3 ± 4.1	11.5 ± 3.9	12.7 ± 3.7*	10.2 ± 3.8	9.3 ± 4.2*	11.4 ± 4.5
K-LCQ total	11.1 ± 3.2	10.9 ± 3.0	10.4 ± 3.2*	12.1 ± 2.8*	12.6 ± 3.5*	11.1 ± 3.1
Physical domain	4.1 ± 1.0	4.0 ± 0.9	3.7 ± 1.0*	4.4 ± 0.8*	4.5 ± 1.1*	4.1 ± 0.7
Psychological domain	3.5 ± 1.2	3.4 ± 1.1	3.2 ± 1.1*	3.8 ± 1.1	3.9 ± 1.3*	3.4 ± 1.2
Social domain	3.6 ± 1.3	3.5 ± 1.2	3.4 ± 1.4	3.9 ± 1.2	4.1 ± 1.6*	3.6 ± 1.4

*UACS* upper airway cough syndrome, *CVA* cough variant asthma, *EB* eosinophilic bronchitis, *GERD* gastro-esophageal reflux disease, *NRS* numeric rating scale, *COAT* COugh Assessment Test, *K-LCQ* Korean version of Leicester Cough Questionnaire.

*Indicate statistical significance (*p* < 0.05).

### Characteristic features of chronic cough according to causes

**Figure 1:**
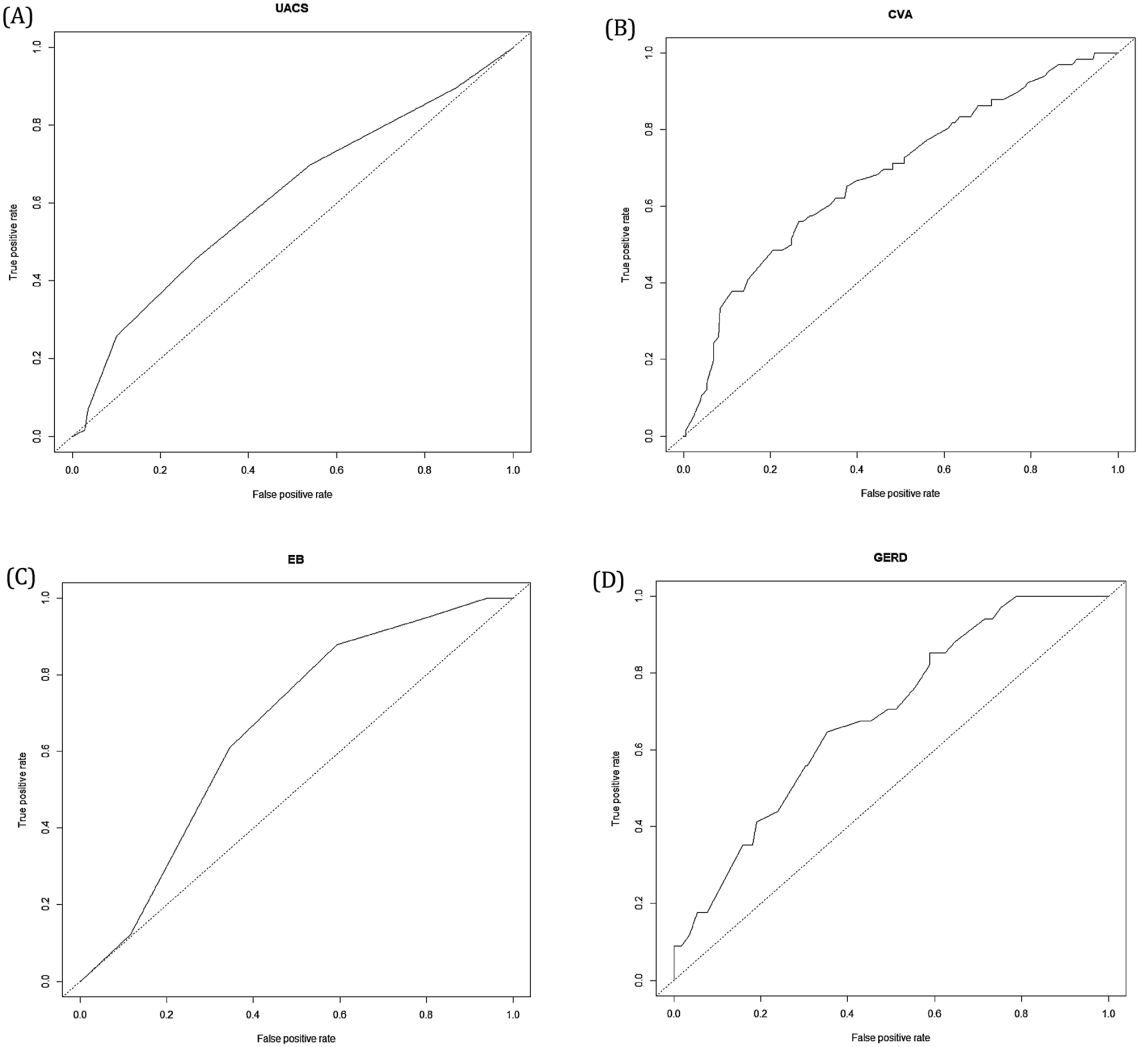
Receiver operating curve logistic regression for diagnosing upper airway cough syndrome (**A**), asthma/cough variant asthma (**B**), eosinophilic bronchitis (**C**), and gastro-esophageal reflux disease (**D**). Area under curve of the receiver operating characteristics curve (AUC) of UACS, CVA, EB, and GERD: 0.60, 0.71, 0.70, 0.77, respectively.

Using the K-LCQ questionnaire, subjects with UACS presented with more voice hoarseness. Subjects with asthma/CVA complained of more bothersome phlegm, tiredness, hypersensitivity to irritants, and sleep disturbance in physical domain; feeling out of control with cough and embarrassment in psychological domain; interference with job/daily task or life enjoyment in social domain. Those with EB showed less chest/stomach pain, bothersome phlegm, and hoarseness in physical domain; less worry about serious illness in psychological domain; less job/daily activity or life enjoyment interference in social domain. Subjects with GERD also presented with less phlegm, tiredness and hoarseness in physical domain; less frustration, feeling fed up, and concern about other’s thoughts in psychological domain; and less interference with job/daily task or life enjoyment in social domain. Patients with multiple causes or idiopathic cough showed no clinical difference with those with single cause. Detailed scores of respective items of K-LCQ and their comparisons are summarized in Table 2. For multivariable analysis, stepwise-logistic regression was performed for each disease. Since K-LCQ measures cough-specific quality of life that higher score indicates better quality of life with lesser symptoms, therefore, odds ratios less than 1 represent correlation with severe symptoms with lesser scores for QOL; odds ratios more than 1 relate to lesser symptoms with higher scores for QOL. In UACS, more hoarseness of voice was chosen (OR 0.76). Female sex (OR 2.16), more tiredness (OR 0.79), and hypersensitivity to irritants (OR 0.82) were significantly associated with asthma/CVA. For EB, less voice hoarseness (OR 1.59), and for GERD, less feelings of being fed-up (OR 1.35) as well as less voice hoarseness (OR 1.42) were selected. Multivariable model for idiopathic cough was not able to be built. Results of the multivariable analysis are summarized at Table 3. The AUC of ROC curve for classification of UACS, asthma/CVA, EB, and GERD were 0.60, 0.71, 0.70, and 0.77, respectively (Fig. 1). The model of logistic regression was validated using LOOCV and predictive validity was 0.76, 0.82, 0.87, and 0.89 for UACS, asthma/CVA, EB, and GERD.

**Table 2:** Comparison of different phenotype among different causes of chronic cough using K-LCQ.

	Items	Total (N = 246)	UACS (N = 115)	Asthma/CVA (N = 65)	EB (N = 40)	GERD (N = 31)	Idiopathic (N = 16)
**Physical**							
1	Chest/stomach pain	4.7 ± 1.4	4.6 ± 1.5	4.6 ± 1.5	5.2 ± 1.1*	4.7 ± 1.4	4.8 ± 1.1
2	Bothersome phlegm	3.6 ± 1.7	3.4 ± 1.7	3.1 ± 1.6*	4.1 ± 1.9*	4.3 ± 1.8*	4.1 ± 1,7
3	Tiredness	3.9 ± 1.5	3.9 ± 1.6	3.4 ± 1.5*	4.2 ± 1.4	4.50 ± 1.6*	4.1 ± 1.6
9	Hypersensitivity to irritants	4.1 ± 1.5	4.0 ± 1.6	3.7 ± 1.6*	4.4 ± 1.3	4.6 ± 1.5	4.1 ± 1.4
10	Sleep disturbance	4.2 ± 1.5	4.2 ± 1.5	3.9 ± 1.5*	4.3 ± 1.4	4.7 ± 1.5	4.3 ± 1.3
11	Coughing bout frequency	3.8 ± 1.3	3.7 ± 1.3	3.6 ± 1.3	4.1 ± 1.3	4.1 ± 1.3	3.8 ± 1.3
14	Voice hoarseness	4.9 ± 1.4	4.6 ± 1.5*	4.6 ± 1.5	5.6 ± 1.0*	5.5 ± 1.1*	5.1 ± 1.2
15	Loss of energy	3.3 ± 1.5	3.3 ± 1.5	3.0 ± 1.5	3.6 ± 1.4	3.6 ± 1.5	2.9 ± 1.5
**Psychological**							
4	Feeling in cough of control	2.7 ± 1.3	2.6 ± 1.2	2.4 ± 1.2*	3.0 ± 1.3	2.7 ± 1.4	2.7 ± 1.2
5	Embarrassment	3.6 ± 1.4	3.6 ± 1.2	3.3 ± 1.3*	3.9 ± 1.2	4.2 ± 1.7	3.5 ± 1.4
6	Anxiety	3.1 ± 1.4	3.1 ± 1.3	2.9 ± 1.4	3.2 ± 1.4	3.5 ± 1.7	3,4 ± 1,4
12	Frustration	4.4 ± 1.5	4.4 ± 1.5	4.2 ± 1.4	4.7 ± 1.5	5.0 ± 1.4*	4.2 ± 1.7
13	Feeling of fed-up	3.3 ± 1.6	3.2 ± 1.6	3.1 ± 1.5	3.6 ± 1.6	4.1 ± 1.6*	3.1 ± 1.6
16	Worries about serious illness	3.5 ± 1.6	3.4 ± 1.6	3.3 ± 1.5	4.0 ± 1.4*	3.8 ± 1.9	3.3 ± 1.5
17	Concern other people’s thought	3.6 ± 1.6	3.5 ± 1.5	3.4 ± 1.5	3.9 ± 1.6	4.3 ± 1.9*	3.2 ± 1.7
**Social**							
7	Job/daily activity interference	3.7 ± 1.5	3.7 ± 1.4	3.3 ± 1.5*	4.2 ± 1.3*	4.3 ± 1.6*	3.9 ± 1.7
8	Overall life enjoyment interference	3.6 ± 1.4	3.5 ± 1.4	3.2 ± 1.4*	4.1 ± 1.3*	4.2 ± 1.5*	3.4 ± 1.3
18	Interruption of conversation/phone call	3.9 ± 1.6	3.9 ± 1.5	3.9 ± 1.6	4.0 ± 1.6	4.2 ± 1.8	3.9 ± 1.6
19	Annoyance to partner/friend/family	3.2 ± 1.7	3.1 ± 1.6	3.1 ± 1.7	3.5 ± 1.6	3.9 ± 2.2	3.1 ± 1.6
Total LCQ		11.1 ± 3.2	10.9 ± 3.0	10.4 ± 3.2*	12.1 ± 2.8*	12.6 ± 3.5*	11.1 ± 3.1

*K-LCQ* Korean version of Leicester Cough Questionnaire, *UACS* upper airway cough syndrome, *CVA* cough variant asthma, *EB* eosinophilic bronchitis, *GERD* gastro-esophageal reflux disease.

*Indicate statistical significance (*p* < 0.05).

**Table 3:** Multivariable analysis and relative impact of each item on cause of chronic cough.

	Item	Odds ratio	95% CI
UACS	Voice hoarseness (LCQ 14)	0.76	0.54–0.91
Asthma/CVA	Female sex	2.16	1.12–4.17
	Tiredness (LCQ3)	0.79	0.64–0.96
	Hypersensitivity (LCQ9)	0.82	0.69–0.99
EB	Voice hoarseness (LCQ14)	1.59	1.19–2.11
GERD	Feeling of fed-up (LCQ13)	1.35	1.05–1.74
	Voice hoarseness (LCQ14)	1.42	1.01–1.99

*UACS* upper airway cough syndrome, *CVA* cough variant asthma, *EB* eosinophilic bronchitis, *GERD* gastro-esophageal reflux disease, *LCQ* Leicester cough questionnaire, *CI* confidence interval.

**Figure 2:**
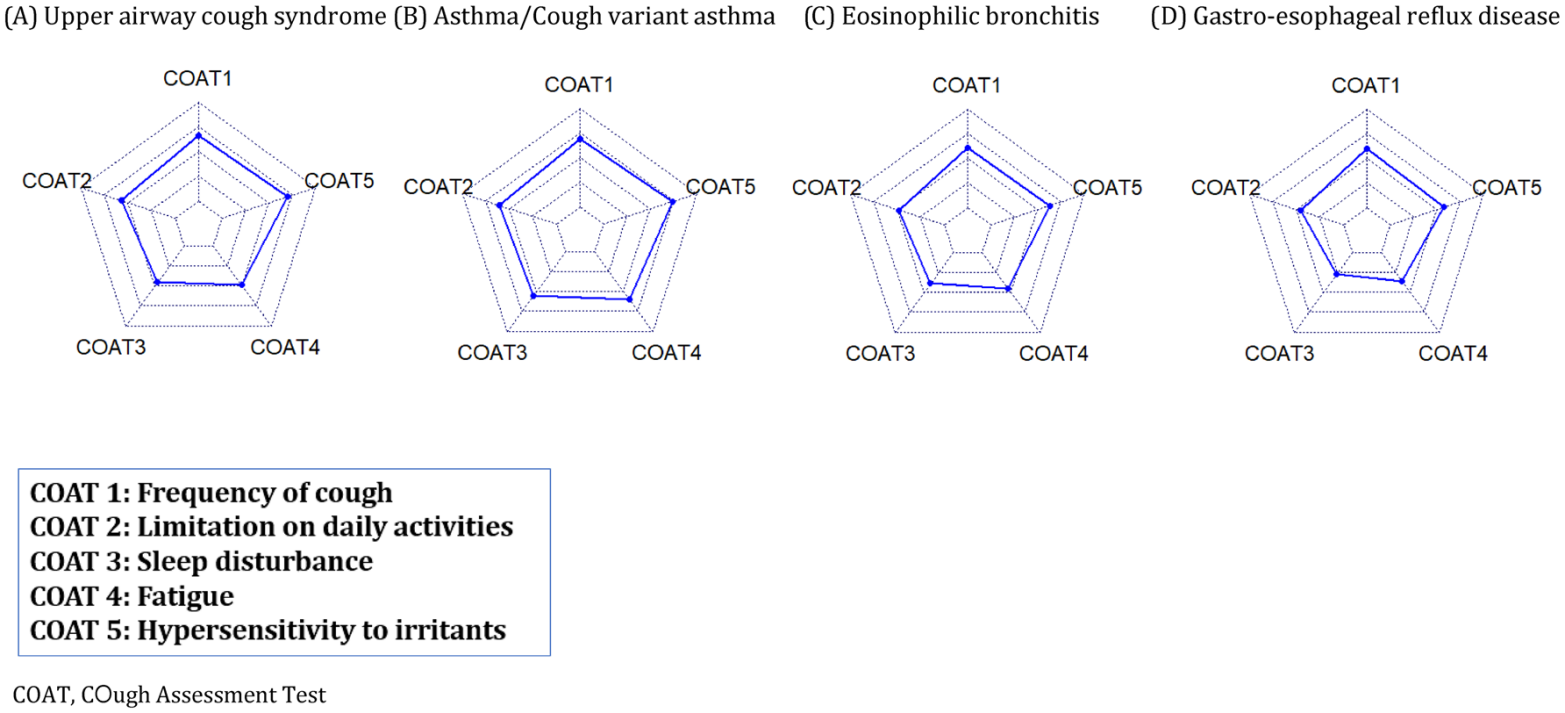
Comparison of score of each item of COAT among each cause of chronic cough. (**A**) Upper airway cough syndrome, (**B**) Asthma/Cough variant asthma, (**C**) Eosinophilic bronchitis, (**D**) Gastro-esophageal reflux disease. *COAT* COugh Assessment Test.

Subsequently, detailed scores of each item of COAT questionnaire are described in Supplemental Table S1. In addition, radar charts comparing the patterns of COAT for each cause are drawn in Fig. 2. In multivariable logistic regression using the COAT questionnaire, no item was selected for UACS. For asthma/CVA, female sex (OR 2.33, p = 0.01) and sleep disturbance (OR 1.54, p = 0.001) were selected. Less daily activity limitation (OR 0.74, p = 0.06) for EB, and less sleep disturbance (OR 0.62, p = 0.005) and less hypersensitivity to irritants (OR 0.68, p = 0.03) for GERD were selected. However, AUC of asthma/CVA, EB, and GERD were 0.68, 0.59, and 0.70, which was not sufficiently high.

### Decision tree predicting the causes of chronic cough

**Figure 3:**
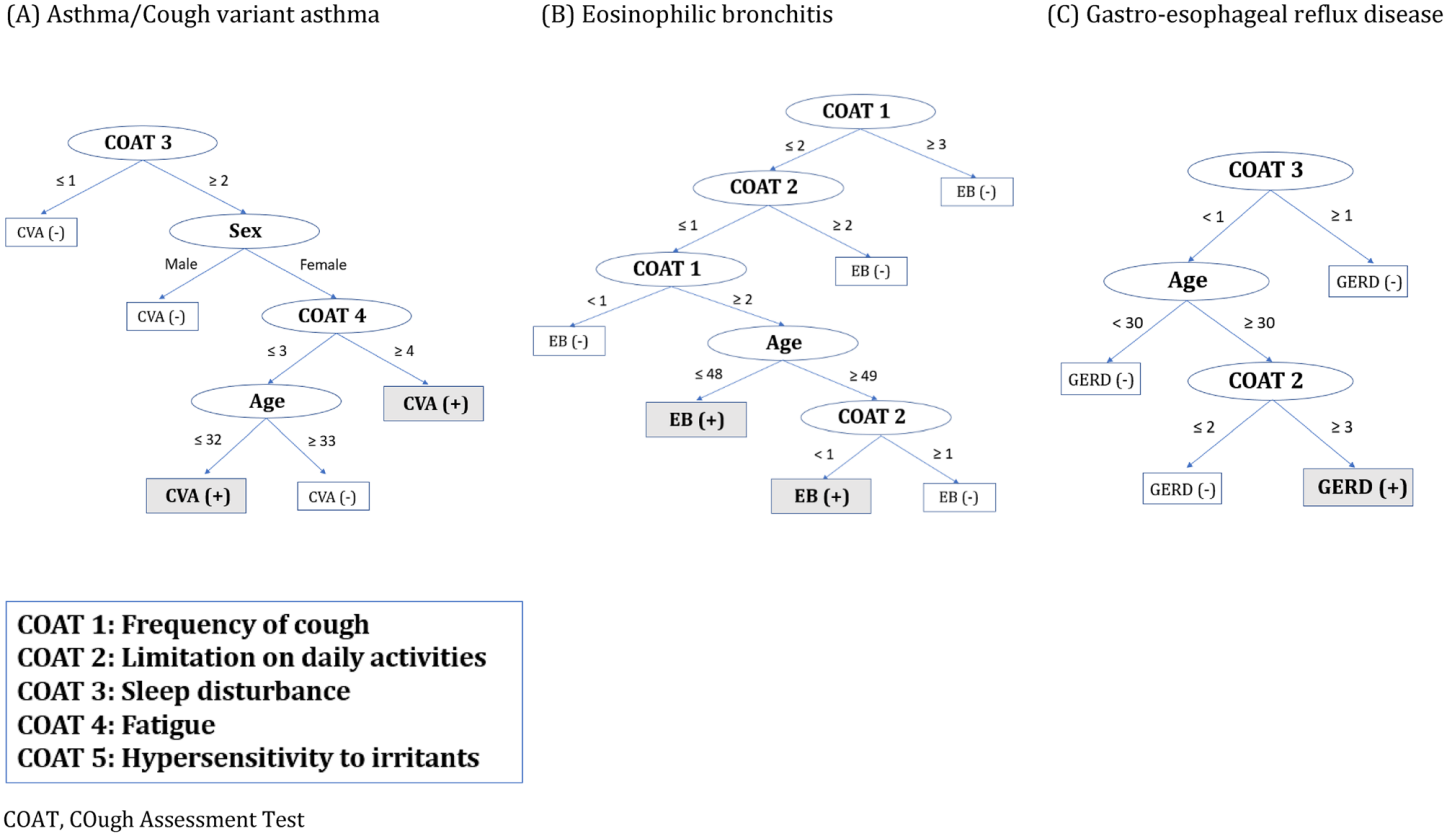
Decision tree for (**A**) asthma/cough variant asthma, (**B**) eosinophilic bronchitis, and (**C**) gastroesophageal reflux disease using COAT questionnaire. *COAT* COugh Assessment Test.

A decision tree was constructed to examine the non-parametric model. Initially, an integrated decision tree to diagnose all causes at once was built using items of K-LCQ in addition to age, sex, and current smoking status; LCQ items 2 (phlegm), 3 (tiredness), 5 (embarrassment), 9 (hypersensitivity to irritants), 10 (sleep disturbance), 15 (loss of energy), and 17 (concern for others), age, and sex were selected (Supplemental Fig. S4A). However, the accuracy of this decision tree was only 0.50. When we modeled another decision tree using the components of COAT, factors 2 (daily activity limitation), 3 (sleep disturbance), 4 (fatigue), age, sex, and current smoking status were selected (Supplemental Fig. S4B). Nevertheless, the accuracy of this decision tree was also low with 0.49. Therefore, a specific decision tree for each cause of chronic cough was re-constructed using K-LCQ or COAT score. For UACS, a decision tree using K-LCQ selects item 2 (phlegm) and 14 (hoarseness), and accuracy of the tree using these 2 items was 0.60 (Supplemental Fig. S5A). Using COAT items, item 2 (daily activity limitation), 4 (fatigue), age and current smoking were chosen, and accuracy was 0.64 (Supplemental Fig. S6). In case of asthma/CVA, a decision tree using K-LCQ selected item 1 (chest/stomach pain), 3 (tiredness), 5 (embarrassment), 15 (loss of energy), 16 (worries about serious illness) and age, and the accuracy was 0.80 (Supplemental Fig. S5B). Using COAT, factor 3 (sleep disturbance), 4 (fatigue), age and sex were selected (Fig. 3A), and accuracy was 0.76. In EB, K-LCQ item 1 (chest/stomach pain), 2 (phlegm), 3 (tiredness), 7 (job/activity interference), 9 (hypersensitivity to irritants), 10 (sleep disturbance), 14 (voice hoarseness) and 19 (annoyance to partner/friend/family) were selected for K-LCQ tree (Supplemental Fig. S5C), and accuracy was 0.88. For COAT tree, factor 1 (cough frequency), 2 (daily activity limitation) and age were selected (Fig. 3B), and accuracy was 0.83. In GERD, K-LCQ item 1 (chest/stomach pain), 5 (embarrassment), 9 (hypersensitivity to irritants), 13 (feeling fed-up), 19 (annoyance to partner/friend/family) and current smoking status were selected for K-LCQ tree (Supplemental Fig. S5D), and COAT factor 2 (daily activity limitation), 3 (sleep disturbance) and age were selected for COAT tree (Fig. 3C); their accuracy was 0.89 and 0.85, respectively.

## Discussion

In this study, we characterized and compared the features of chronic cough according to underlying causes using commonly available cough questionnaires. Patients with asthma/CVA suffered from more severe cough, especially in physical and psychological domain. Meanwhile, patients with GERD showed less severity in all 3 domains. Despite the different severities of cough in each cause, the time to visit a hospital was similar. Remarkably, voice hoarseness was an independent feature of UACS, supposed to be caused by post-nasal drip or inflammation on larynx located nearby. Female sex and hypersensitivity to irritants were predictors for asthma/CVA, consistent with previous description^18,19^. Less hoarseness was characteristics of EB and feeling fed-up and hoarseness were less common features of GERD; less severe cough may less likely induce secondary laryngitis. Based on the differential manifestation according to etiology, we built a practical decision trees to predict the causes. Moreover, the classification tree using COAT—simplified version questionnaire—showed similar accuracy to that of K-LCQ, which suggests that this COAT algorithms could be easily applied to everyday clinical practice.

Most of the previous studies on chronic cough have been focused on prevalence, identifying common causes, or development of effective diagnostic flow for laboratory tests^2–17^. However, a description of the detailed features and comparison of characteristics to distinguish each etiology had been limited, which is a fundamental step in medical practice. Our study revealed the characteristic differences of chronic cough by each etiology. One of interesting findings is the differences between asthma/CVA and EB. Although they share immunopathology of eosinophilic airway inflammation^20^, the detailed features between them have not been characterized. Patients of chronic cough with EB were not female dominant; less severe features; and specific differences of each item in the cough questionnaire were observed, unlike those with asthma/CVA. Furthermore, present study attempted to make a simple classification algorithm to decide which test should proceed further using clinical features. The decision tree enables physicians to classify causes very fast that can serve as a useful tool in clinical practice. Also, decision trees may provide clues for the pathogenesis of disease due to their own structure. Initially, building a single decision tree for every cause was tried, but accuracy of this classification was unsatisfactory. This supposed to be from multiple causes accompanied by, therefore, separate decision trees for each cause were re-constructed. These decision trees, specific to each cause, produced higher accuracy except for UACS; which suggested that the reason for low accuracy at initial single tree might be attributable to UACS. Since a simple physical examination could enhance the diagnostic accuracy of UACS, further large studies including this information are needed.

Several limitations should be addressed. Detailed results of physical examination, spirometry, bronchoprovocation test, eosinophil count in induced sputum, or exhaled fraction of nitric oxide were not reviewed in this analysis. Therefore, there could be some possibilities of misdiagnosis that underestimate the accuracy of our model. Nevertheless, the clinical diagnosis was decided by pulmonary specialists in respiratory centers, considering their available facilities. Validation of the COAT is tested only in a single country^21^ and would need to be further generalized. Though we tried to use cross-validation in our analysis, we did not have external validation set to test our model. Since prevalence of chronic cough could be different among different countries, predictive performance of decision tree could be lower if practitioners in different countries use different diagnostic criteria particularly in primary care setting. Therefore, ascertainment of this cohort may limit generalizability to the other races. Further large studies to confirm our findings are needed, especially from different countries. Lastly, information about comorbidities could have enhance the understanding of their relation to symptoms and pathophysiology.

In conclusion, the degree of tiredness, hypersensitivity to irritants, feeling fed-up, voice hoarseness, and sex are important features in determining etiologies of chronic cough, and the simplified COAT questionnaire can be used to distinguish causes as well as measurement of cough severity. Further large studies to confirm our findings are necessary.

## Methods and materials

### Study subjects

Adult patients (≥ 18 years old) with chronic cough lasting more than 8 weeks were recruited from 16 respiratory centers in Korea from March 1, 2016 to February 28, 2018. All the possible candidates were enrolled during these periods. The possible cause of chronic cough was assessed via the diagnostic flow of Korean cough guideline by pulmonary specialists in each hospital, excluding those with suspected abnormalities on chest radiography^15^. Enrolled participants completed both the Korean version of the Leicester Cough Questionnaire (K-LCQ) and the COugh Assessment Test (COAT) (Supplemental Fig. S1). The K-LCQ is a validated cough-specific quality of life (QOL) questionnaire containing 19 items divided into 3 domains: physical, psychological, and social^22,23^. A 7-point Likert scale is used to evaluate the responses for each item, and the total scores are calculated by summation of the mean converted values of each domain, which range from 3 to 21; higher score indicates better quality of life. Physical domain section includes questions about chest/stomach pain, accompany of bothersome phlegm, tiredness, hypersensitivity to irritants, sleep difficulties, frequency of coughing bouts, presence of voice hoarseness, and loss of energy due to cough. In psychological domain, questions of feeling fed-up, worrying about serious illness, and concerns of what other people might think are included. Social domain contains questions of interference with job or daily tasks, life enjoyment, interruption of telephone call conversation, and annoyance of partner, family, or friend. The COAT is a simplified version of the K-LCQ and used to assess the severity of cough composed of 5 factors: frequency of cough, limitation on daily activities, sleep disturbance, fatigue, and hypersensitivity to irritants^21^. All factors are scored on a single scale ranging from 0 to 4 (total scores from 0 to 20), where a higher score means more severe cough. Consequently, K-LCQ and COAT scores are highly associated with negative direction^21^. This study was conducted in accordance with Declaration of Helsinki and was approved by the Institutional Review Boards (IRB) of Ilsan Paik Hospital, Republic of Korea. Exemption of informed consent was also obtained from IRB.

### Statistical analysis

All the statistical analysis was performed using R version 3.6.0. Patients’ characteristics are presented as mean (± standard deviation) or median (quartiles) for continuous variables and relative frequencies for categorical variables. Means were compared using t-test or analysis of variance and categorical variables were compared using chi-squared test. A *p*-value < 0.05 was considered to be statistically significant. For multivariable analysis, logistic regression was performed to diagnose each cause using all items of questionnaire in addition to age, sex and current smoking status; the best classification model was selected by stepwise selection method using stepAIC function in MASS package. Stepwise regression is step-by-step iterative construction of a regression model to select predictive variables by automatic procedure. In each step, potential explanatory variables are added or subtracted from the previous model and tested for statistical significance after each iteration based on prespecified criteria: Akaike Criterion. To compare the diagnostic ability of each model, area under curve (AUC) of the receiver operating characteristics curve (ROC) was calculated using ROCR package. To validate predictive power of logistic regression, leave-one-out cross validation (LOOCV) was performed using boot package^24^. To make a decision tree for the prediction of each cause, tree package was used. Tree is a nonparametric statistical procedure containing classification by using a set of if–then-else logical conditions to assign unknown features to a predefined category. Algorithms for constructing tree work are from top to down, by choosing a variable at each step that best splits the set of items^25^. Tree creates partition recursively to increase purity in the direction to lower the impurity using Gini index. The training set and test set was divided into 7:3 ratio for cross validation, and decision tree was modeled at train set with all items of K-LCQ or COAT questionnaire in addition to age, sex, and current smoking status. Number of pruning nodes was selected by K-fold cross validation, and accuracy of tree model was validated at test set. Venn diagram and radar chart were drawn using venn Diagram function in limma package and radarchart function in fmsb package, respectively.

## Supplementary Information

Supplementary Information.

## Abbreviations

AUC: Area under curve
COAT: COugh Assessment Test
CVA: Cough variant asthma
EB: Eosinophilic bronchitis
GERD: Gastroesophageal reflux syndrome
K-LCQ: Korean version of the Leicester Cough Questionnaire
QOL: Quality of life
ROC: Receiver operating characteristics curve
UACS: Upper airway cough syndrome
