# Supplementary material for: Differential features of chronic cough according to etiology and the simple decision tree for predicting causes
Source: Sci Rep. 2021 May 14;11:10326. doi: 10.1038/s41598-021-89741-z (PMC8121949; doi:10.1038/s41598-021-89741-z)
Supplement: Supplementary file 1 — Supplementary Information. [file 41598_2021_89741_MOESM1_ESM.pdf]

Differential features of chronic cough according to etiology and the simple decision tree for predicting causes

Short title: Decision tree for causes of cough

Authors

Hyeon-Kyoung Koo[1], Won Bae[1], Ji-Yong Moon[2], Hyun Lee[3], Jin Woo Kim[4], Seung Hun Jang[5], Hyoung Kyu Yoon[6], Deog Kyeom Kim[7*]

Supplements

Supplemental Table S1. Comparison of different phenotype among different causes of chronic cough using COAT

	Item	Total (N = 236)	UACS (N = 116)	Asthma/CVA (N = 66)	EB (N = 41)	GERD (N = 34)	Idiopathic (N = 16)
1	**Cough frequency**	2.61 ± 0.82	2.64 ± 0.82	2.75 ± 0.77	2.43 ± 0.81	2.48 ± 0.93	2.56 ± 0.89
2	**Daily activity limitation**	2.22 ± 1.04	2.28 ± 0.90	2.42 ± 1.06	1.93 ± 1.07	1.87 ± 1.18*	2.38 ± 1.15
3	**Sleep disturbance**	1.76 ± 1.21	1.80 ± 1.17	2.20 ± 1.15*	1.58 ± 1.08	1.13 ± 1.12*	1.50 ± 1.32
4	**Fatigue**	1.98 ± 1.19	1.96 ± 1.15	2.39 ± 1.13*	1.80 ± 1.16	1.45 ± 1.18*	1.81 ± 1.33
5	**Hypersensitivity to irritants**	2.78 ± 1.00	2.79 ± 1.00	2.92 ± 1.02	2.50 ± 1.01	2.36 ± 1.05*	3.19 ± 0.75
**Total COAT**		11.35 ± 4.06	11.46 ± 3.86	12.68 ± 3.74*	10.50 ± 3.84	9.26 ± 4.12*	11.44 ± 4.53

Abbreviations: COAT, Cough Assessment Test; UACS, upper airway cough syndrome; CVA, cough variant asthma; EB, eosinophilic bronchitis; GERD, gastro-esophageal reflux disease

* Indicate statistical significance (*P*<0.05)

Supplemental Figure S1. COugh Assessment Test (COAT)

How severe do you cough? Please complete the survey below

Following survey is to assess the extent of cough and its disturbance to your daily life, both physically and mentally. Within 3 days, how did the following problems affect you? Please check that best fits with you.

	Not at all	Occasionally	Somewhat	Very much	Extremely
**How frequent did you cough?**	⓪	①	②	③	④
**How much did coughing affect your daily life?**	⓪	①	②	③	④
**How bothersome was the cough on your sleep?**	⓪	①	②	③	④
**How easily did you feel fatigued by the cough?**	⓪	①	②	③	④
**Did you cough more often when in dust, smelling irritant odor, or inhaling cold air?**	⓪	①	②	③	④
**Total score**					

Supplemental Figure S2. Correlation matrix between each item of COAT and K-LCQ questionnaire

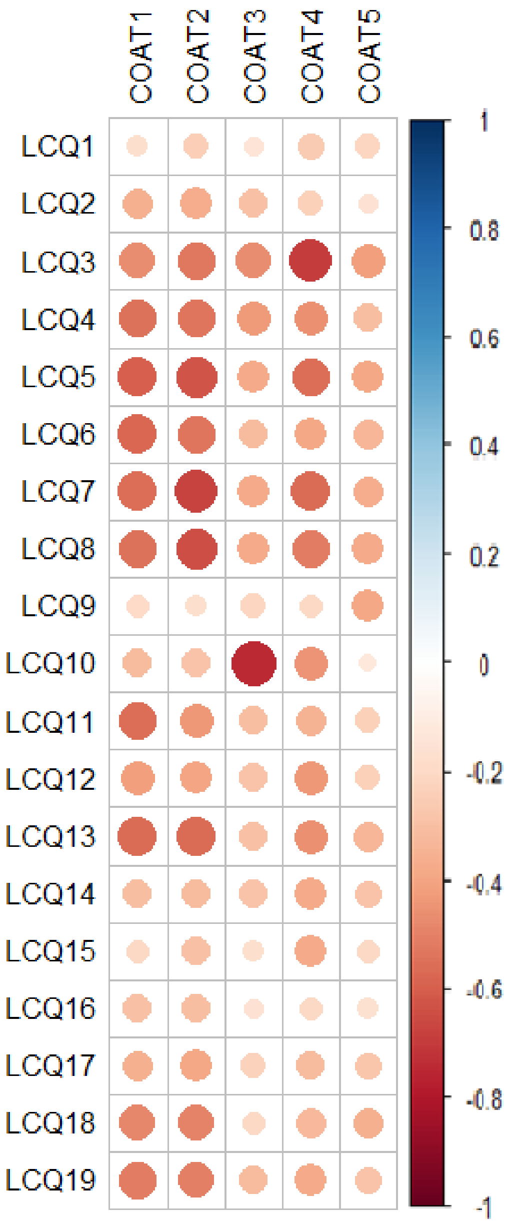

Supplemental Figure S3. Distribution of causes for chronic cough

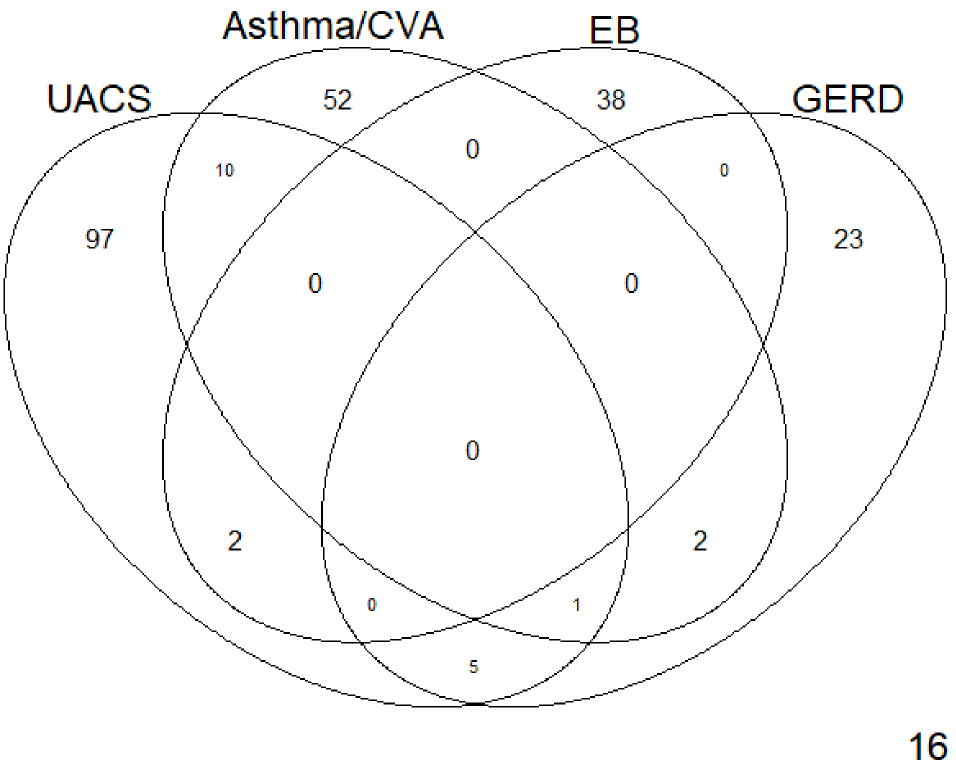

UACS, upper airway cough syndrome; CVA, cough variant asthma; EB, eosinophilic bronchitis; GERD, gastro-esophageal reflux disease

Supplemental Figure S4. Decision tree for diagnosing cause of chronic cough using (A) K-LCQ and (B) COAT

(A) Accuracy: 0.504

(B) Accuracy: 0.492

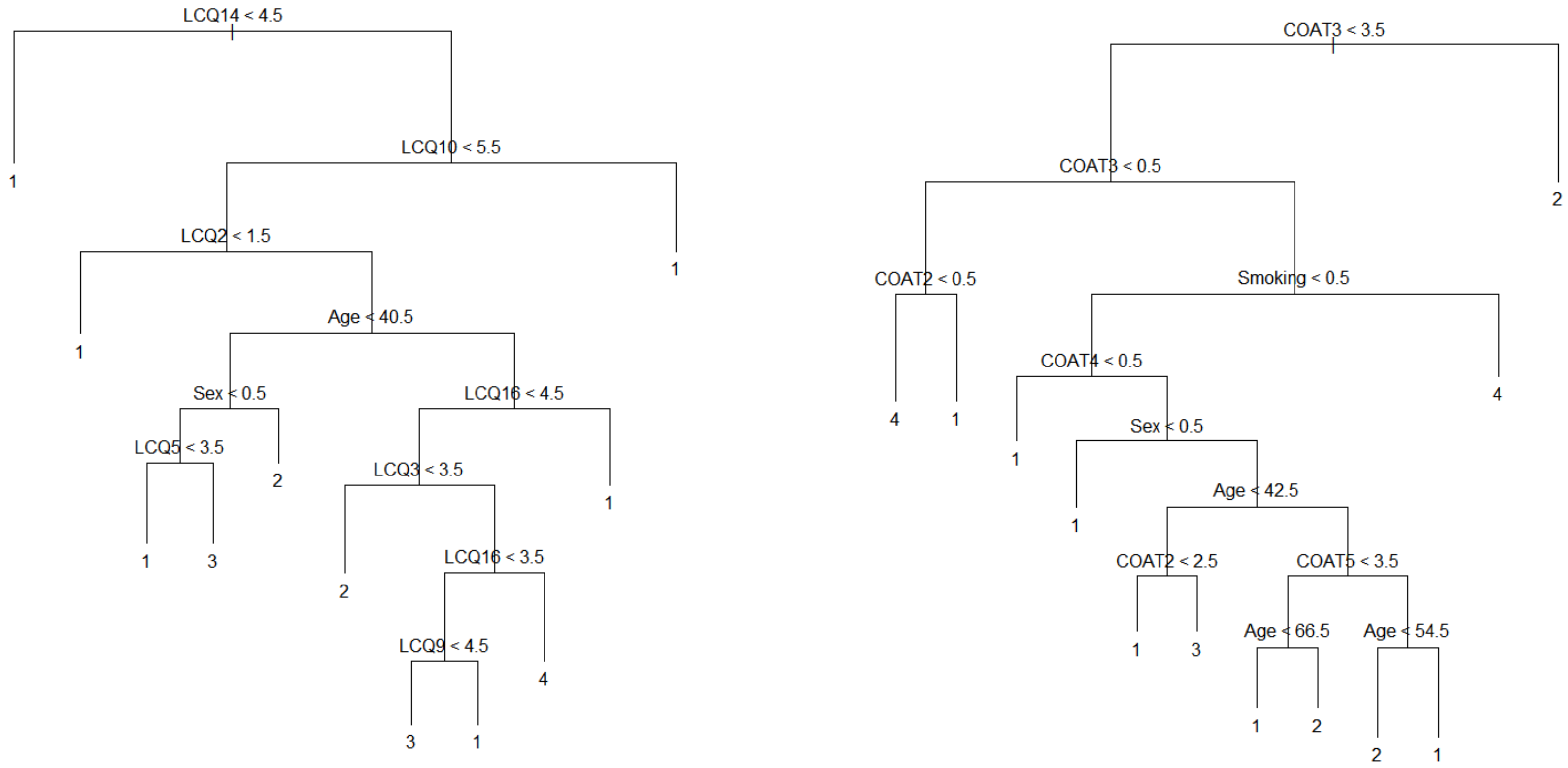

At each node, “Yes” goes to left sided edge and “No” goes to right sided edge.

1, Upper airway cough syndrome; 2, Asthma/Cough variant asthma; 3, Eosinophilic bronchitis; 4, Gastroesophageal reflux disease

Supplemental Figure S5. Decision tree for (A) upper airway cough syndrome, (B) cough variant asthma, (C) eosinophilic bronchitis, and (D) gastroesophageal reflux disease using K-LCQ

(A) Accuracy: 0.60

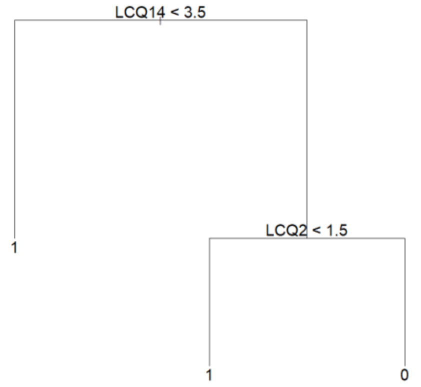

(B) Accuracy: 0.80

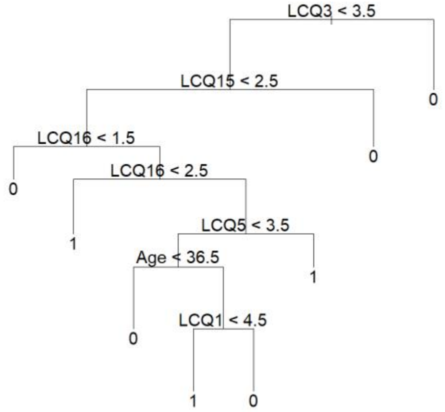

(C) Accuracy: 0.88

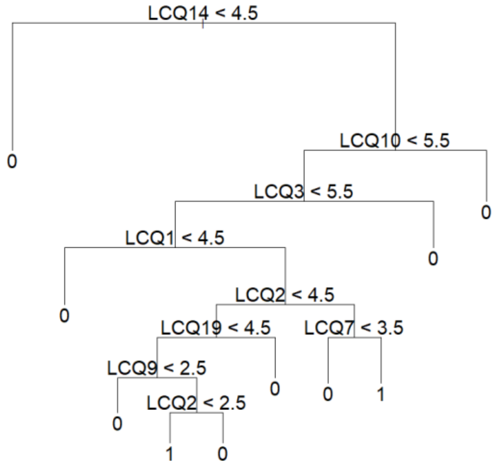

(D) Accuracy: 0.89

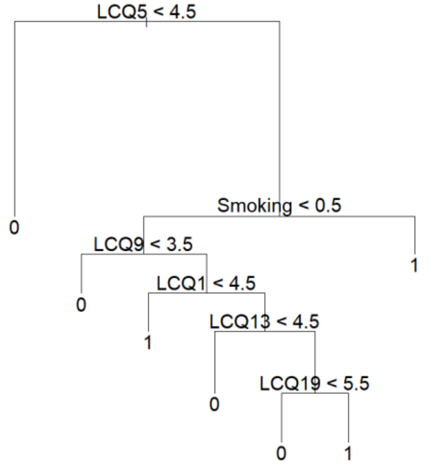

At each node, “Yes” goes to left sided edge and “No” goes to right sided edge

Physical: 1. Chest/stomach pain; 2. Bothersome phlegm; 3. Tiredness; 9. Hypersensitivity to irritants; 10. Sleep disturbance; 11. Coughing bout frequency; 14. Voice hoarseness; 15. Loss of energy

Psychological: 4. Feeling in cough of control; 5. Embarrassment; 6. Anxiety; 12. Frustration; 13; Feeling of fed-up; 16. Worries about serious illness; 17. Concern other people’s thought

Social: 7. Job/daily activity interference; 8. Overall life enjoyment interference; 18. Interruption of conversation/phone call; 19. Annoyance to partner/friend/family

Supplemental Figure S6. Decision tree for upper airway cough syndrome using COAT

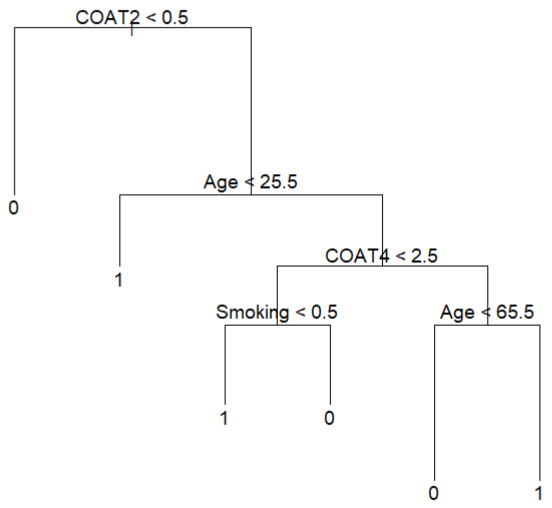

At each node, "Yes" goes to left sided edge and "No" goes to right sided edge.
